# Supplementary material for: Spectroscopic glimpses of the transition state of ATP hydrolysis trapped in a bacterial DnaB helicase
Source: Nat Commun. 2021 Sep 6;12:5293. doi: 10.1038/s41467-021-25599-z (PMC8421360; doi:10.1038/s41467-021-25599-z)
Supplement: Supplementary file 1 — Supplementary Information [file 41467_2021_25599_MOESM1_ESM.pdf]

Supplementary Information

Spectroscopic glimpses of the transition state of ATP hydrolysis trapped in a bacterial DnaB helicase

Alexander A. Malär[1], Nino Wili[1], Laura A. Völker[1], Maria I. Kozlova[2], Riccardo Cadalbert[1], Alexander Däpp[1], Marco E. Weber[1], Johannes Zehnder[1], Gunnar Jeschke[1], Hellmut Eckert[3,4], Anja Böckmann[5], Daniel Klose[1,*], Armen Y. Mulkidjanian[2,6,*], Beat H. Meier[1,*] and Thomas Wiegand[1,7,8*]

[1] *Physical Chemistry, ETH Zurich, 8093 Zurich, Switzerland*

[2] *School of Bioengineering and Bioinformatics and Belozersky Institute of Physico-Chemical Biology, Lomonosov Moscow State University, 119234, Moscow, Russia*

[3] *Institut für Physikalische Chemie, WWU Münster, 48149 Münster, Germany*

[4] *Instituto de Física de Sao Carlos, Universidad de Sao Paulo, Sao Carlos SP 13566-590, Brazil*

[5] *Molecular Microbiology and Structural Biochemistry UMR 5086 CNRS/Université de Lyon, 69367 Lyon, France*

[6] *Department of Physics, Osnabrueck University, 49069 Osnabrueck, Germany*

[7] *present address: Max-Planck-Institute for Chemical Energy Conversion, Stiftstr. 34-36, 45470 Mülheim an der Ruhr, Germany*

[8] *present address: Institute of Technical and Macromolecular Chemistry, RWTH Aachen University, Worringerweg 1, 52074 Aachen, Germany*

*Corresponding authors: daniel.klose@phys.chem.ethz.ch, armen.mulkidjanian@uni-osnabrueck.de, beme@ethz.ch, thomas.wiegand@phys.chem.ethz.ch

Supplementary Figures and Tables

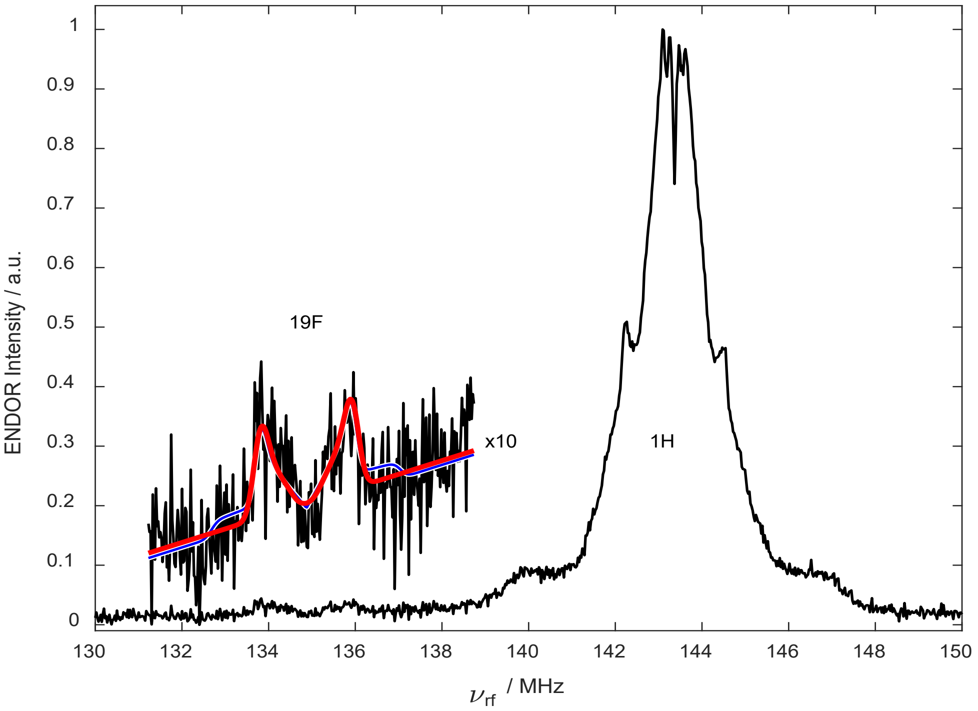

Supplementary Figure 1: Davies ENDOR recorded on DnaB:ADP:AlF_4^-, in a spectral region that features ^1H and ^{19}F transitions. Many intense and relatively strongly coupled protons are visible, as well as one or more relatively weakly coupled ^{19}F nuclei. This is consistent with the EDNMR spectra. Here, the ENDOR spectrum was acquired with stochastic rf excitation, the inversion pulse length was set to 400 ns, and the echo was integrated over a time of 800 ns (see the Methods section for further parameters). The inset shows a zoom into the ^{19}F region (black) and two simulations: blue: A_{iso} = 0, T = 2.19 MHz, red: A_{iso} = -1.62, T = 0.61 MHz. The simulations explicitly included the blind spot behavior of Davies ENDOR around A = 0 MHz and a linear baseline correction. The signal-to-noise ratio (SNR) is not sufficient to distinguish the two models. Assuming the point-dipole approximation, the hyperfine anisotropies T yield electron spin-^{19}F distances of 3.3 Å (blue) or 5.0 Å (red), respectively. However, analysis of the anisotropic hyperfine couplings to ^{19}F can be complicated by spin density in the ^{19}F p-orbitals, which may invalidate the point-dipole approximation, and hence these distances should be considered as rough estimates, especially at the present SNR. The signals clearly indicate that ^{19}F is in proximity of the Mn(II) ion.

Supplementary Table 1: Experimental and calculated (in brackets) ^{31}P hyperfine coupling tensor values. The models used for the DFT calculations are shown in Supplementary Figure 1. δ denotes the anisotropy and η_A the asymmetry of the hyperfine coupling tensor. [a] calculated values based on the *Bst*DnaB model [b] calculated values based on the *Aa*DnaB model.

	A_{iso}/ MHz	A_{xx}/ MHz	A_{yy}/ MHz	A_{zz}/ MHz	δ/ MHz	$\lvert\eta_A\rvert$
^{31}Pα	0.3 (0.05^a, 0.01^b)	0.23 (-0.24^a, -0.32^b)	0.23 (-0.23^a, -0.28^b)	0.37 (0.61^a,0.64^b)	0.14 (0.85^a, 0.66^b)	0.00 (0.02^a, 0.06^b)
^{31}Pβ	4.7 (3.35^a,12.8^b)	3.8 (2.36^a,11.3^b)	3.8 (2.63^a,11.5^b)	6.4 (5.07^a,15.6^b)	2.6 (2.58^a, 4.20^b)	0.00 (0.16^a, 0.07^b)

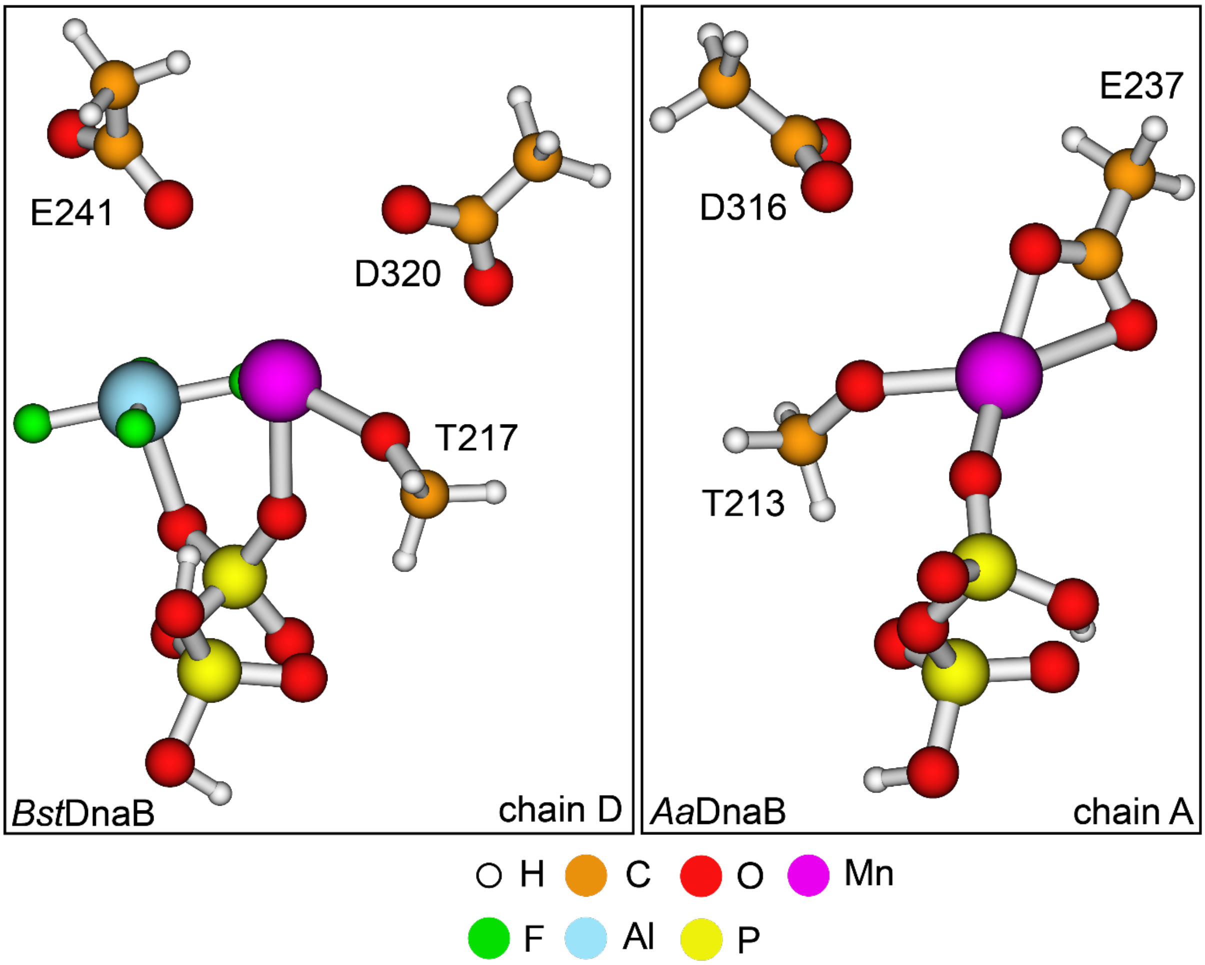

Supplementary Figure 2: Models extracted from the PDB files 4ESV (*Bst*DnaB) and 4NMN (*Aa*DnaB) used in the DFT calculations of the hyperfine coupling tensors. The pictures have been created with the software VMD (version 1.9.3)[1].

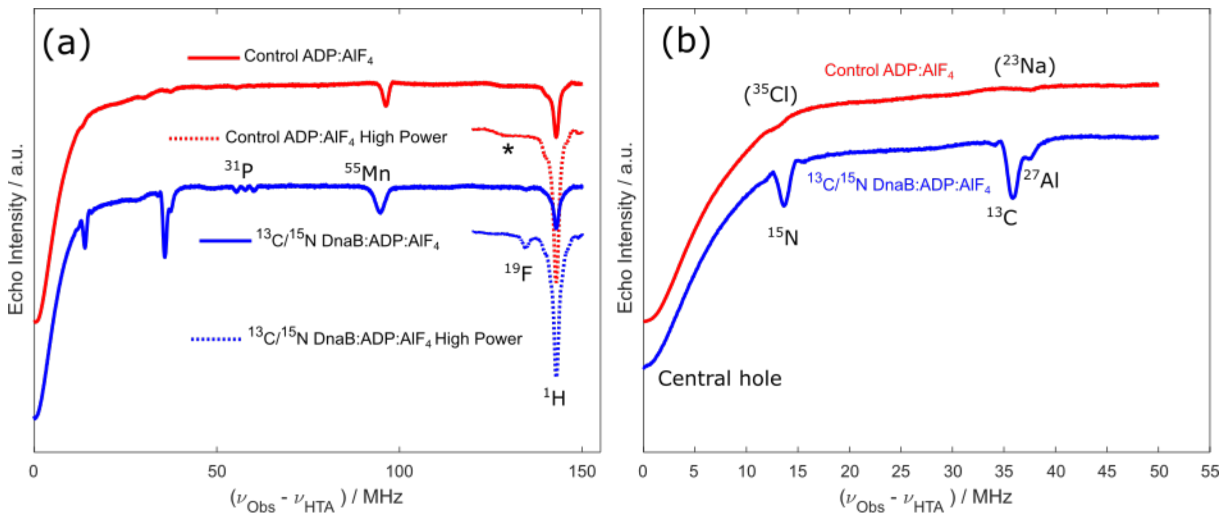

Supplementary Figure 3: **The binding of the metal ion cofactor and ADP:AlF$_4^-$ to DnaB revealed by EDNMR experiments performed on ^{13}C/^{15}N-labeled DnaB.** (a) EDNMR spectra using low and high HTA-pulse power, are shown with solid and dotted lines, respectively. The spectra of a control solution containing only Mn^{2+}:ADP:AlF$_4^-$ in the same buffer used for the protein sample (red) show no specific peaks due to hyperfine interaction, only contributions near the baseline from the buffer constituents ^{23}Na and ^{35}Cl, as well as peaks from ^{1}H, and ^{55}Mn (see annotation). The * marks a broad, currently unassigned, feature that could either be due to Mn double quantum transitions or combination lines[2], or simply a baseline artefact due to the high power HTA pulses. In contrast, the spectrum of Mn^{2+}:DnaB:ADP:AlF$_4^-$ (blue) using ^{13}C/^{15}N-labeled DnaB shows clear peaks around the ^{13}C and ^{15}N nuclear Zeeman frequencies, as annotated in the zoom into the low-frequency region in (b). These arise due to many ^{13}C and ^{15}N nuclei with small hyperfine couplings to the electron spin on Mn^{2+}. In addition a peak at the ^{27}Al frequency shows concomitant proximity of ^{27}Al. Taken together these observations reveal binding of Mn^{2+} to DnaB.

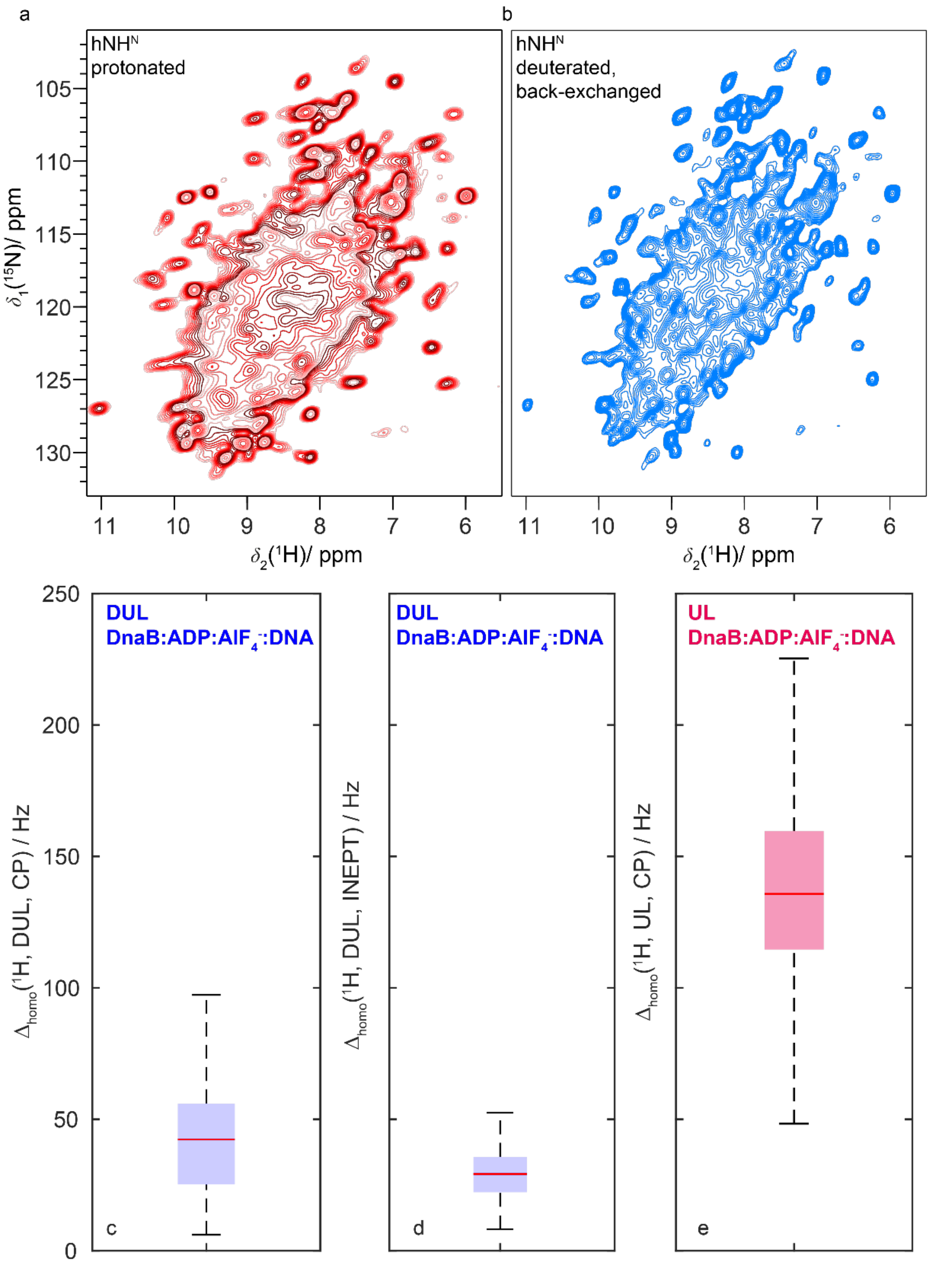

Supplementary Figure 4: The proton-linewidths of the deuterated and 100 % back-exchanged sample decrease compared to a fully-protonated sample. 2D hNH correlation spectra of fully protonated DnaB complexed with ADP:AlF_4^-:DNA (**a**, taken from reference [3], Creative Commons CC BY) and deuterated and 100% back-exchanged DnaB in complex with ADP:AlF_4^-:DNA (**b**).Boxplot representation of homogeneous proton linewidth statistics in DnaB:ADP:AlF_4^-:DNA determined from site-specific $T_2'(^1H)$ measurements on isolated resonances in a 2D hNH spectrum of a deuterated and 100 % back-exchanged sample ((**c**) using CP and **d** using refocused INEPT as a polarization transfer mechanism) and for a fully protonated sample (**e**). Latter have already been reported in reference[3]. The homogeneous proton line-widths roughly decrease by a factor of three in the deuterated compared to the fully-protonated sample. The number of independently analyzed linewidths *n* is 23/47/150, while the

boxplot defining parameters are median=42/29/136 Hz, minimum value = 6/8/48 Hz, maximum value = 97/52/310 Hz, upper whisker = 97/53/225 Hz, lower whisker = 6/8/48 Hz, 25th percentile = 25/22/114 Hz and 75th percentile = 56/36/159 Hz for **c**, **d** and **e** respectively.

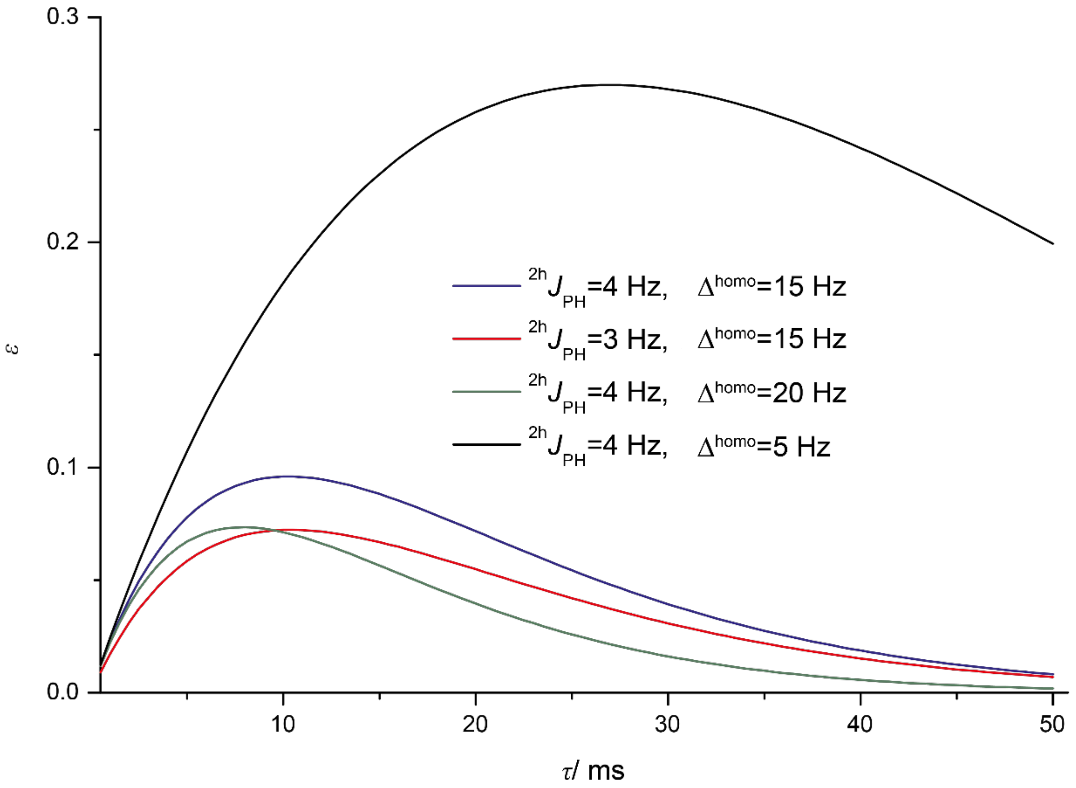

Supplementary Figure 5: INEPT enhancement factor ε as a function of the mixing time assuming different (relatively small) Δ^{homo} values and $^{2h}J_{PH}$ J-coupling constants. Infinitely long ^{31}P transverse relaxation has been assumed for all simulations. The simulations have been performed based on the well-known formulas for an HX spin system[4, 5, 6]. The $^{2h}J_{PH}$ couplings are so weak that even for the smallest homogeneous linewidth of 5 Hz, the theoretical transfer efficiency stays below 30%, while it is already less than 10% in all other cases.

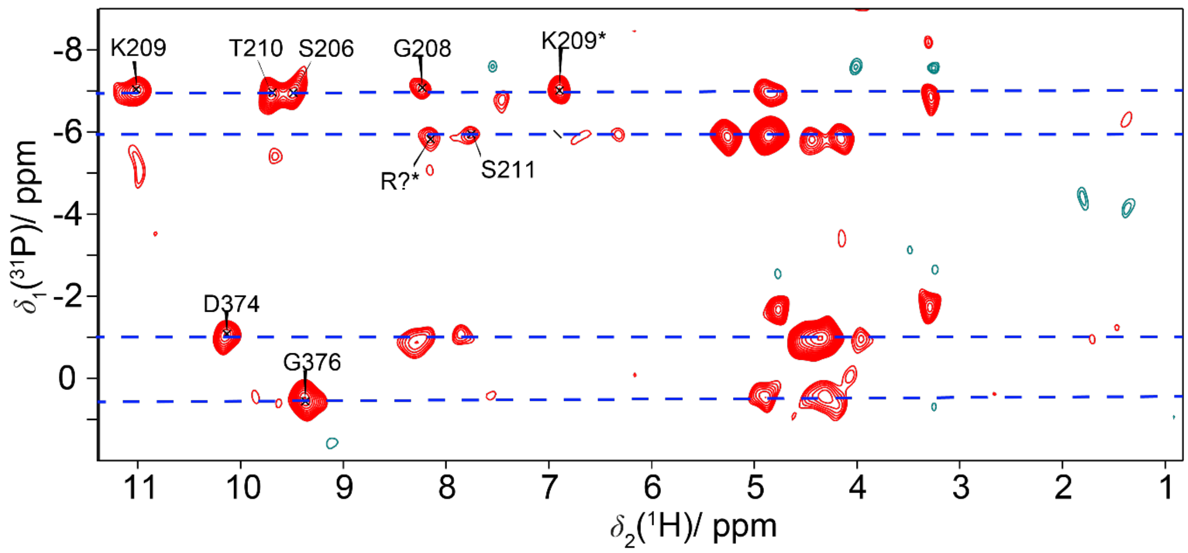

Supplementary Figure 6: hPH correlation spectrum recorded at 20.0 T with a MAS frequency of 105 kHz and using a forward and backward CP-contact time of 1.5 ms. The dashed blue lines indicate the chemical-shift positions of the four ^{31}P resonances from Figure 3a. * stands for correlations to side-chain protons.

Residue	Average distance/ Å
N-O3B S213 (S206)	3.0
N-O2B G215 (G208)	3.1
N-O2B K216 (K209)	2.4
NZ-O3B K216 (K209)	2.7
N-O1B T217 (T210)	3.3
N-O1A/O2A A218 (S211)	3.1

Supplementary Table 2: Distances between the oxygen phosphate groups of GDP and protein nitrogen atoms in the *Bst*DnaB crystal structure (PDB:4ESV). The residues shown in brackets correspond to the ones of *Hp*DnaB. The average distance for the five bound GDP molecules is given. Note, that for A218 the closest distance to one of the two oxygen atoms O1A and O2A has been chosen.

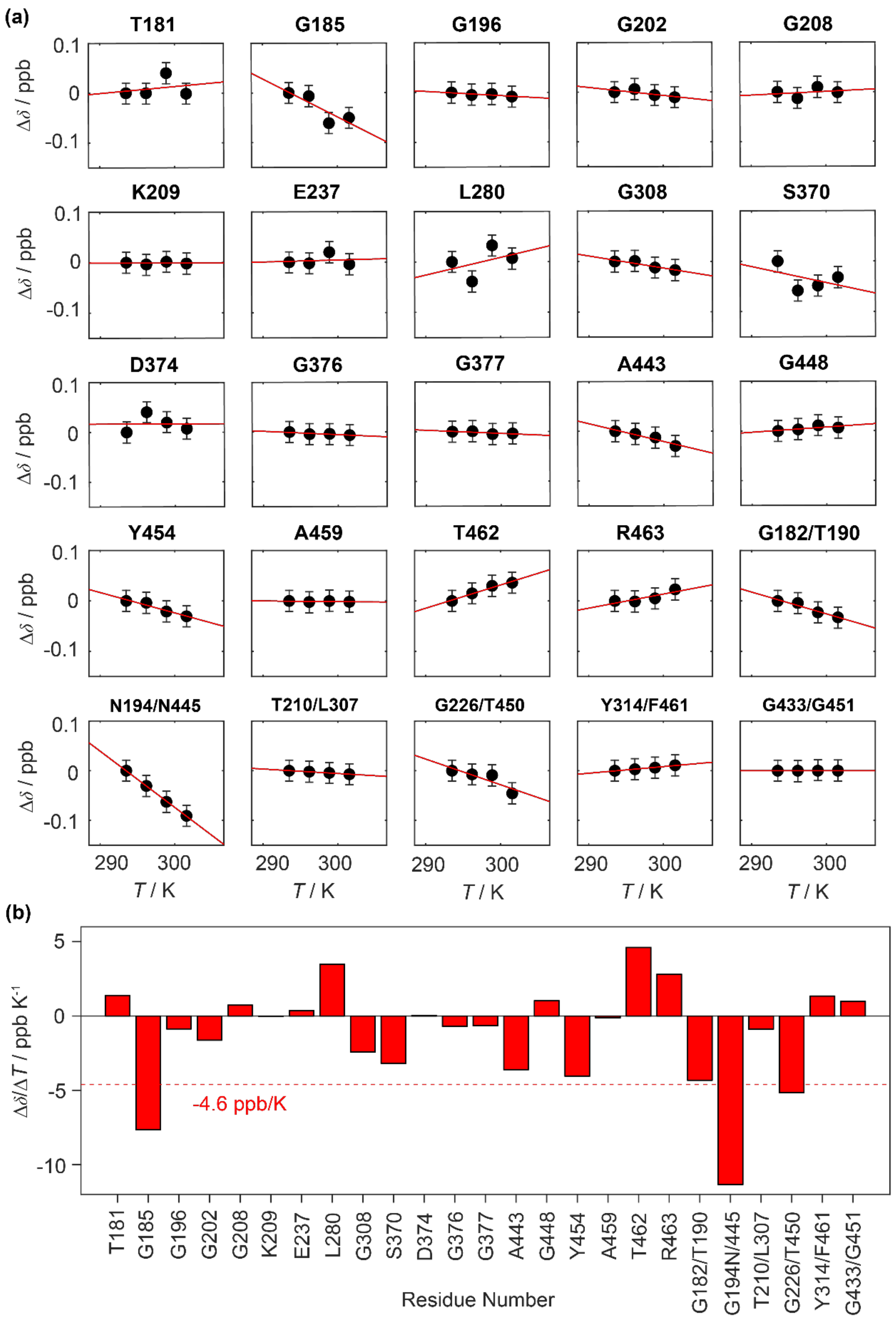

Supplementary Figure 7: **Proton chemical-shift temperature coefficients. (a)** Residue-specific temperature-dependent proton chemical-shift values (black circles) between 294-302 K and linear fit (red) for the extraction of correspondent temperature coefficients. The chemical shifts are referenced to the corresponding value at 294 K. Chemical-shift values were extracted from n=1 experiments and are represented as $\delta \pm 0.05$ ppm, where the shown error bar

represents an estimate of the expected uncertainty within such experiments. **(b)** Site-specific proton chemical-shift temperature coefficients determined from the slope of the linear regression of the data points shown in **(a)**.

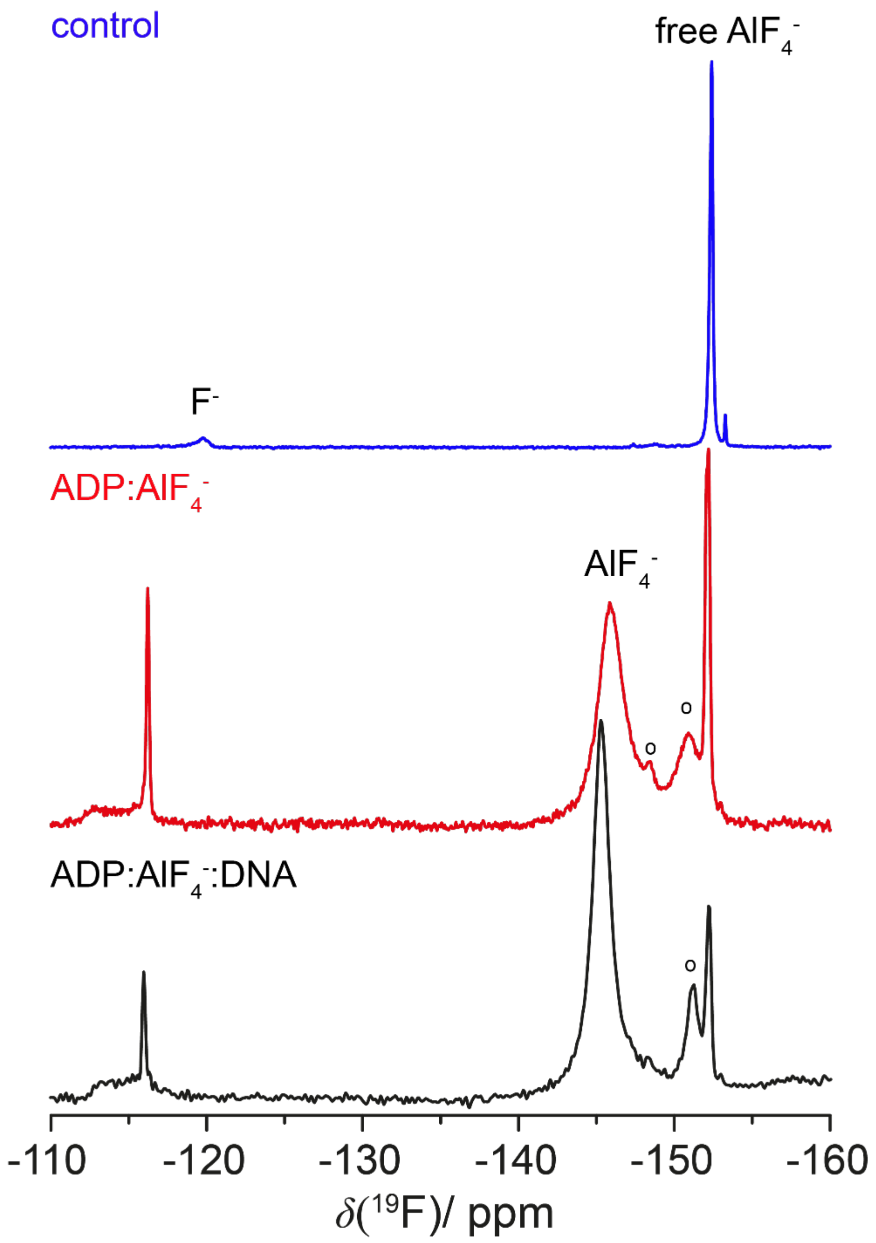

Supplementary Figure 8: ^{19}F MAS spectra indicate the fast rotation of the AlF_4^- unit. ^{19}F MAS spectra recorded at 14.1 T with an MAS frequency of 17.0 kHz and with the EASY background suppression scheme[7]. Spectra were acquired on DnaB:ADP:AlF_4^- in the presence and absence of DNA, as well as on the buffer solution without protein (control experiment). o indicates precipitated $AlF_x(OH)_{6-x}$ species.

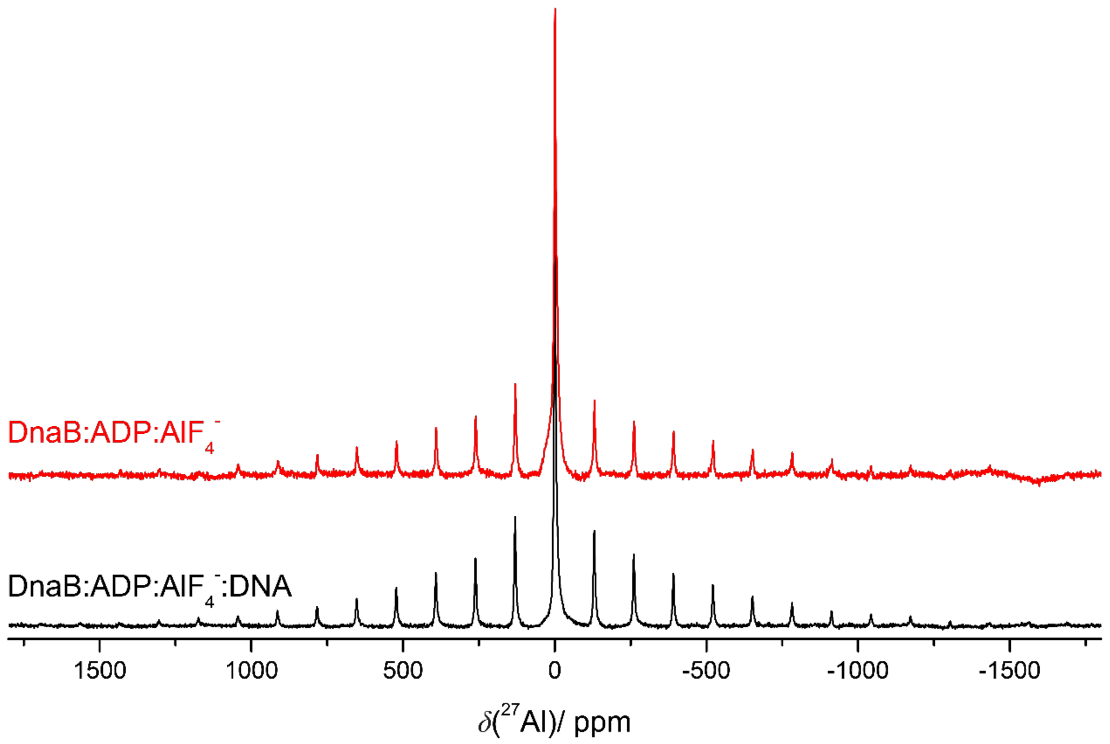

Supplementary Figure 9: **The AlF$_4^-$ unit is mobile in absence and presence of DNA.** ^{27}Al MAS spectra of DnaB:ADP:AlF$_4^-$ and DnaB:ADP:AlF$_4^-$:DNA recorded at 11.7 T and 17 kHz MAS.

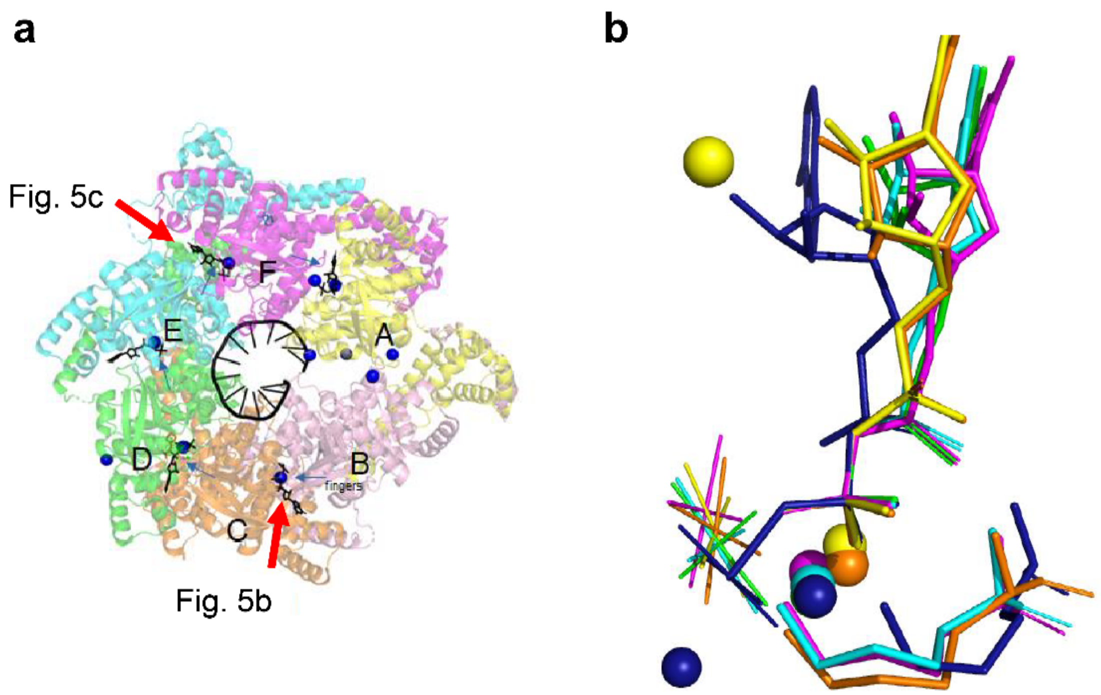

Supplementary Figure 10: Relative orientation of the AlF_4^- moieties in all five occupied binding sites of the DnaB structure from *Geobacillus stearothermophilus* (*Bst*DnaB, see also Figure 5, PDB accession code 4ESV and reference [8]). The colors of AlF_4^- moieties (**b**) correspond to the colors of subunits to which they are bound (**a**). For comparison, the dark-blue colored structure of the ADP:AlF_4^- : H_2O_{cat} complex from the ABC ATPase of the maltose transporter MalK (see PDB accession code 3PUW and reference [9]) is shown. NDPs were superimposed by atoms O^{3A}, P^B and O^{3B} in Pymol[10].

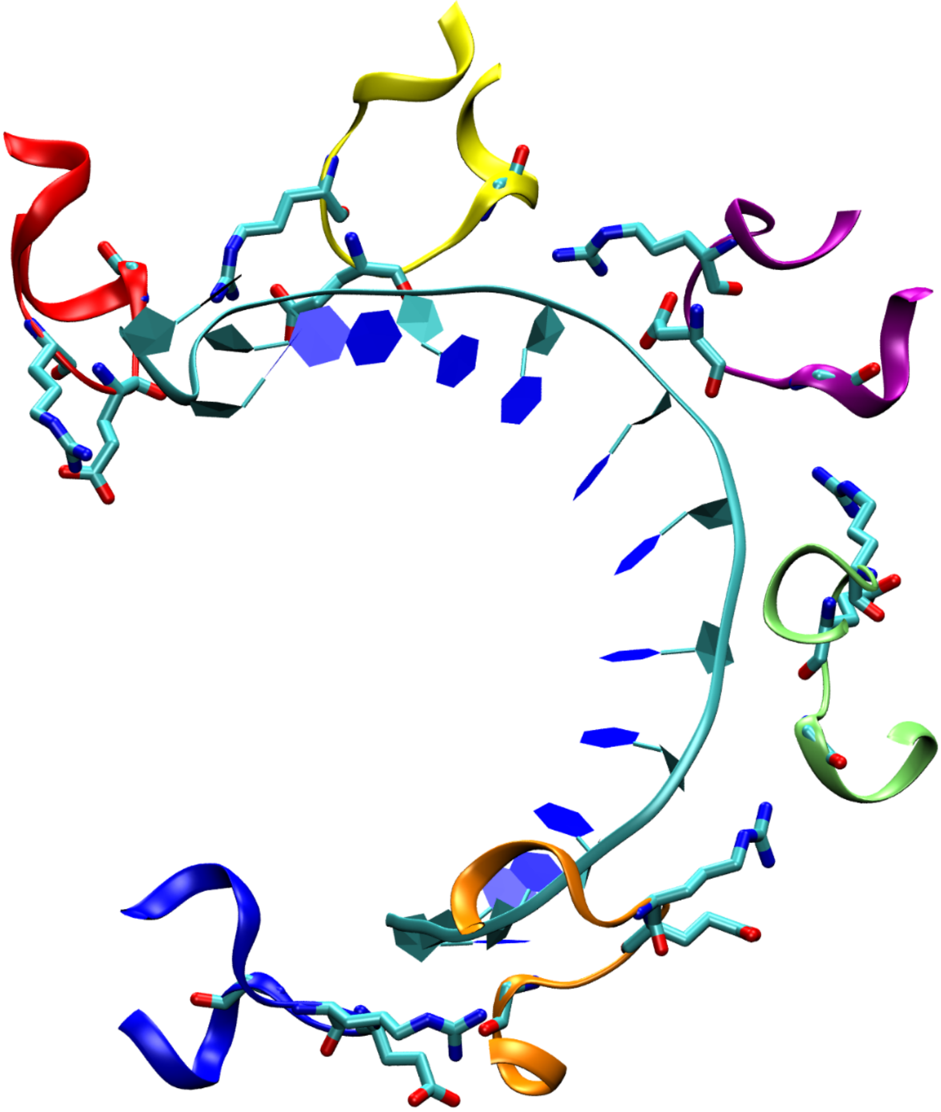

Supplementary Figure 11: **DNA binding in BstDnaB.** DNA binding in the *Bst*DnaB:DNA complex (PDB 4ESV). The DNA binding loops (residues 378-387) are shown in different colors. Residues R381, E382 and G384 are shown in licorice style. The DNA is shown in ribbon representation.

Supplementary Note 1: Additional mechanistic considerations of P-loop NTPases.

The reported free rotational diffusion of all six AlF_4^- moieties within the tight hexamer of *Hp*DnaB (Figure 5) could be rationalized within the general mechanistic framework of P-loop NTPases. Their catalytic cycle provides energy for mechanical work during the two of its several catalytic steps. First, upon the exergonic binding of a NTP molecule to a P-loop domain, the binding energy is used to re-organize the catalytic site, which includes pulling two or three protein loops with electrostatically compensating positive charges closer to the triphosphate moiety; the relocations of these loops cause conformational changes that can be used for doing useful work. For instance, binding of ATP drives the largest 65° step in the rotation of the central stalk of F1-ATPase[11]. Specifically, in *Hp*DnaB the energy of ATP binding is used to bind the NBD domain to the DNA strand which results in unwinding of the double helix.

During the next step, a specific activating partner interacts with the catalytic site. This interaction is accompanied by insertion of a stimulating moiety/moieties into the catalytic site and its closure. Within the closed catalytic site, the NTP hydrolysis takes place. In diverse NTPases, the tight binding of transition state analogues, such as ADP:AlF_4^-, ensures closing of the catalytic pocket, engagement of stimulating moiety/moieties and formation of a transition-state-like configuration[12, 13, 14]. In the case of *Hp*DnaB, the hydrolysis is stimulated by Lys444 and Arg446 of the neighbouring monomer (Figure 7).

Although the catalytic bond to γ-phosphate breaks upon hydrolysis, the resulting $H_2PO_4^-$ anion stays initially in the catalytic pocket, being bound by few hydrogen bonds[11, 15]. The subsequent phosphate release is again exergonic; the departure of $H_2PO_4^-$, owing to its interactions with particular side-chains on the way out, opens the catalytic pocket and can drive large-scale conformational changes; the phosphate escape, for instance, drives the power stroke step in myosins, for which a set of structures with the moving away $H_2PO_4^-$ is available[15]. In the case of SF4 helicases, and, specifically *Bst*DnaB, the phosphate release is believed to be coupled with the translocation of the helicase subunits along the DNA strand [8].

Supplementary References

1. Humphrey W, Dalke A, Schulten K. VMD: Visual molecular dynamics. *Journal of Molecular Graphics* **14**, 33-38 (1996).
2. Cox N, Lubitz W, Savitsky A. W-band ELDOR-detected NMR (EDNMR) spectroscopy as a versatile technique for the characterisation of transition metal–ligand interactions. *Mol Phys* **111**, 2788-2808 (2013).
3. Wiegand T*, et al.* Nucleotide binding modes in a motor protein revealed by ^{31}P- and ^{1}H-detected MAS solid-state NMR. *ChemBioChem* **21**, 324-330 (2020).
4. Sørensen OW, Ernst RR. Elimination of spectral distortion in polarization transfer experiments. Improvements and comparison of techniques. *J Magn Reson* **51**, 477-489 (1983).
5. Pegg DT, Doddrell DM, Brooks WM, Robin Bendall M. Proton polarization transfer enhancement for a nucleus with arbitrary spin quantum number from n scalar coupled protons for arbitrary preparation times. *J Magn Reson* **44**, 32-40 (1981).
6. Coelho C, Azaïs T, Bonhomme-Coury L, Laurent G, Bonhomme C. Efficiency of the Refocused 31P−29Si MAS-J-INEPT NMR Experiment for the Characterization of Silicophosphate Crystalline Phases and Amorphous Gels. *Inorg Chem* **46**, 1379-1387 (2007).
7. Jaeger C, Hemmann F. EASY: A simple tool for simultaneously removing background, deadtime and acoustic ringing in quantitative NMR spectroscopy—Part I: Basic principle and applications. *Solid State Nucl Magn Reson* **57–58**, 22-28 (2014).
8. Itsathitphaisarn O, Wing Richard A, Eliason William K, Wang J, Steitz Thomas A. The Hexameric Helicase DnaB Adopts a Nonplanar Conformation during Translocation. *Cell* **151**, 267-277 (2012).
9. Oldham ML, Chen J. Snapshots of the maltose transporter during ATP hydrolysis. *Proc Natl Acad Sci* **108**, 15152-15156 (2011).
10. The PyMOL Molecular Graphics System VS, LLC.
11. Bason JV, Montgomery MG, Leslie AGW, Walker JE. How release of phosphate from mammalian F₁-ATPase generates a rotary substep. *Proc Natl Acad Sci* **112**, 6009-6014 (2015).
12. Wittinghofer A. Signaling mechanistics: Aluminum fluoride for molecule of the year. *Current Biology* **7**, R682-R685 (1997).
13. Jin Y, Richards NG, Waltho JP, Blackburn GM. Metal Fluorides as Analogues for Studies on Phosphoryl Transfer Enzymes. *Angew Chem Int Ed* **56**, 4110-4128 (2017).
14. Shalaeva D, Cherepanov D, Galperin MY, Mulkidjanian AY. Comparative analysis of active sites in P-loop nucleoside triphosphatases suggests an ancestral activation mechanism. *bioRxiv*, 439992 (2018).
15. Llinas P*, et al.* How Actin Initiates the Motor Activity of Myosin. *Developmental Cell* **33**, 401-412 (2015).
